# The association between monthly ambient temperatures and ischemic heart disease mortality in the United States: A nationwide ecological analysis, 1999–2020

**DOI:** 10.1371/journal.pgph.0006641

**Published:** 2026-06-18

**Authors:** Hafiz M. Ahmed, Ubaid ur Rehman, Zubian Ahmed, Muhammad Owais, Ehsan Zaib, Muhammad Moeez Mustafa

**Affiliations:** Department of Medicine, Punjab Medical College, Faisalabad Medical University (FMU), Faisalabad, Pakistan; PLOS: Public Library of Science, UNITED STATES OF AMERICA

## Abstract

Ischemic heart disease (IHD) is the leading cause of death in the United States, but the long-term impact of ambient temperature on IHD mortality remains poorly characterized. Existing research has largely examined short-term temperature extremes. This study is among the first to quantify the association between monthly ambient temperature and IHD mortality across the United States over a 22-year period. In this nationwide ecological study, we examined monthly state-level associations between ambient temperature and IHD mortality from 1999–2020. Mortality data were obtained from CDC WONDER and temperature data from the National Centers for Environmental Information. A negative binomial regression with a distributed lag non-linear model estimated the temperature–mortality relationship, adjusting for state, year, and month fixed effects. Subgroup analyses were conducted by age, sex, race, and climate. A strong inverse association between ambient temperature and IHD mortality was observed (Wald p < 0.001). Colder temperatures were associated with higher mortality risk, whereas warmer temperatures were associated with lower mortality risk at the monthly level. The risk was greatest at the coldest observed temperature (–12.8 °F, RR 1.10, 95% CI: 1.05–1.16) and lowest at the warmest temperature (89.2 °F, RR 0.90, 95% CI: 0.86–0.94), reflecting a 20% difference in relative risk across the observed temperature range. Lag effects were strongest in the month of exposure and attenuated thereafter. The model demonstrated a 93.6% reduction in deviance between null and fully adjusted models. Associations were generally consistent across demographic and climate subgroups, though some estimates were imprecise, likely reflecting smaller event counts. This nationwide ecological analysis identifies a significant inverse association between monthly ambient temperatures and IHD mortality in the U.S. at the population level. These findings highlight ambient temperature as an environmental factor associated with IHD mortality and underscore the need for public health interventions to mitigate cardiovascular mortality during prolonged cold periods.

## 1. Introduction

Ischemic heart disease (IHD) is the leading cause of mortality worldwide, with substantial public health and economic implications. In the United States, IHD accounted for nearly 350,000 of the 3 million annual deaths in 2023. Environmental factors, particularly ambient temperature, have emerged as important determinants of cardiovascular mortality. Extreme cold can increase blood pressure, trigger vasoconstriction, and elevate metabolic demand, whereas extreme heat can cause electrolyte imbalance, dehydration, increased cardiac workload, and systemic inflammatory responses, thereby influencing cardiovascular morbidity and mortality [1–5].

The global literature suggests a heterogeneous temperature–mortality relationship. Studies evaluating the effects of short-term (daily) and extreme temperature variations have often reported U- or J-shaped associations, with elevated risks at both extremes of temperature [6–13]. Many of these investigations, however, have focused on general cardiovascular or all-cause mortality. Although the majority converge on the detrimental effect of cold, the role of heat remains more divergent [14,15]. For example, a Mediterranean study found increased emergency cardiovascular hospitalizations during cold spells but no significant effect of heat [16]. Similarly, a long-term U.S. study in elderly populations reported declining heat-related cardiovascular mortality but persistent cold-related effects [17]. Notably, a recent multi-country analysis focusing specifically on IHD mortality found a decreased risk of IHD incidence and mortality with increasing temperatures [18].

Moreover, most existing research has focused on short-term extremes and daily temperature variations, while the long-term influence of monthly mean ambient temperatures on IHD mortality remains less explored. While daily analyses are well-suited to detecting acute cardiovascular responses to temperature extremes, they are susceptible to mortality displacement bias, short-term forward shifts in death among frail individuals that may not reflect net population-level risk. Monthly mean temperatures, by contrast, reflect sustained thermal exposure and are less affected by this bias, providing a complementary perspective on long-term, population-level temperature–IHD associations [19]. The divergent findings regarding heat effects further highlight critical gaps in the literature. It is unclear whether short-term fluctuations translate into meaningful long-term, population-level trends, or whether cold versus heat poses a greater public health threat warranting targeted interventions. In addition, potential effect modification by age, sex, race, and climate within the U.S. remains poorly characterized.

To address these gaps, we conducted a nationwide analysis of monthly mean ambient temperatures and IHD mortality in the U.S. from 1999 to 2020, using high-quality, publicly available mortality and climate data. We further assessed lagged effects and explored subgroup differences by age, sex, race, and climate, with the aim of providing updated evidence to inform public health policy and prevention strategies.

## 2. Materials and methods

### 2.1. Data sources

This study employed an ecological, retrospective, nationwide time-series design. The data on monthly mortality from ischemic heart disease for all 50 U.S. states and Washington, D.C., from 1999-2020 were obtained using CDC WONDER Database (Underlying Cause of Death by Bridged-Race Categories) [20]. The cause of death was identified using ICD-10 codes I20–I25, which included:

- I20: Angina pectoris
- I21: Acute myocardial infarction
- I22: Subsequent myocardial infarction
- I24: Other acute ischemic heart diseases
- I25: Chronic ischemic heart disease

No specific filters were applied for urbanization, weekdays, autopsy, or place of death. Subgroup data were extracted using the filters for sex, age, and race. The population offset was derived from monthly state-level population estimates obtained from CDC WONDER, which are based on U.S. Census Bureau intercensal population estimates bridged to single-race categories. Data were downloaded as standardized CSV files. All records with valid ICD-10 codes I20–I25 and non-missing geographic/month identifiers were included. CDC WONDER applies standardized coding validated by the National Vital Statistics System.

Monthly mean ambient temperatures for each state were obtained from “National Centers for Environmental Information (NCEI) Climate at a Glance: Statewide Time Series” [21]. No record-level linkage was required, as state-level data from CDC WONDER and NCEI were merged by state and month. Investigators had full access to de-identified, publicly available datasets from CDC WONDER and NCEI. No individual-level data were accessed. Two independent reviewers extracted the data, which were subsequently verified by one additional reviewer to ensure accuracy and consistency. The study was conducted in accordance with **STROBE** and **RECORD** guidelines.

### 2.2. Data analysis

We analyzed the association between monthly mean ambient temperature and IHD mortality using a negative binomial regression model. The model was selected to account for potential overdispersion and heterogeneity across states. Fixed effects were included for state, year, and month to control for spatial variation and seasonality, and a natural cubic spline with 4 degrees of freedom was included to adjust for long-term temporal trends. Mortality counts were modeled as the outcome, with the logarithm of the monthly population entered as an offset.

A distributed lag non-linear model (DLNM) framework was used to assess both the non-linear and delayed effects of ambient temperature on IHD mortality. A cross-basis function was defined using natural cubic splines with three degrees of freedom for both ambient temperature and lag dimensions. A maximum lag period of three months was selected a priori to capture both short-term and potential intermediate-term delayed effects of ambient temperature on IHD mortality, including the known lag between influenza infection, which peaks in winter months, and subsequent cardiovascular events. Lags 0, 1, 2, and 3 correspond to 0, 1, 2, and 3 months, respectively. The estimates for association were summarized as relative risks (RRs) with 95% confidence intervals (CIs) across the observed ambient temperature range. The mean ambient temperature across all states (52.8 °F, 11.6 °C) was chosen as the centering point because the minimum mortality temperature coincided with the upper extreme of ambient temperature and could not be used as a stable reference point. Model predictions were generated using the “crosspred” function to obtain overall exposure–response curves and lag–response surfaces.

The overall significance of the temperature-mortality association was assessed using the Wald statistic. The robustness and stability of the model were evaluated using the Akaike Information Criterion (AIC), null and residual deviance, and the dispersion parameter (θ). The dispersion parameter (θ) reported in the results refers to the NB2 parameterization (variance = μ + μ²/θ), as implemented in the MASS package's glm.nb function. Minimal overdispersion confirmed the appropriateness of the negative binomial specification. Sensitivity analyses were performed by varying the degree of freedom for the exposure–response function (df = 4 and 5 instead of 3). These adjustments did not materially change the AIC, confirming the robustness of the main model.

All analyses were performed using R version 4.5.1 (R Foundation for Statistical Computing, Vienna, Austria), using the *dlnm, MASS, splines, dplyr,* and *ggplot2* packages.

### 2.3. Subgroups

To examine whether the temperature-mortality association varied across population characteristics or climate conditions, we conducted pre-specified subgroup analysis for following categories:

- **Age:** < 45 years, 45–64 years, and ≥65 years
- **Sex:** male and female
- **Race:** White, Black or African American, Asian or Pacific Islander, and American Indian or Alaska Native
- **Climate:** cooler versus warmer states

Although this study assessed racial subgroups, the analysis was not designed to examine racial disparities specifically. Race and ethnicity were included as demographic factors to ensure generalizability and transparency. For climate subgroup classification, we used the overall monthly mean ambient temperature across all states during 1999–2020 (52.8 °F, 11.6 °C) as the cutoff. States with monthly mean ambient temperature below this threshold were classified as cooler states, while those above were categorized as warmer states. This threshold was chosen as an operational definition to maintain consistency with the centering point used in the main analysis and was pre-specified accordingly. It does not correspond to an established climatological classification system, and findings from this subgroup should be interpreted with this in mind. All subgroup categories were pre-specified in the study protocol, and no exploratory or post hoc subgroup analyses were performed.

## 3. Results

### 3.1. Overall association

**Fig 1:**
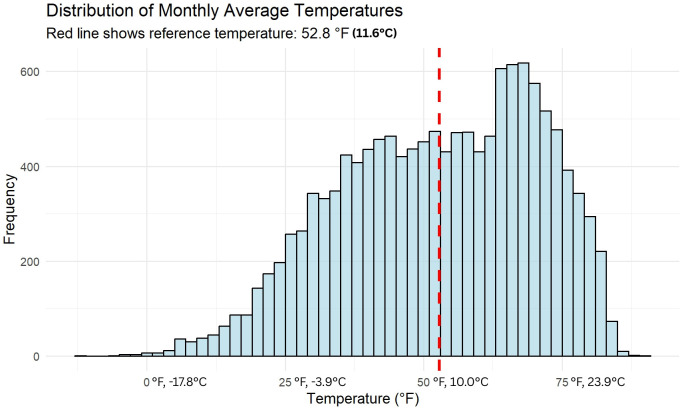
Distribution of monthly mean ambient temperatures across U.S. states, 1999–2020. The histogram displays the frequency distribution of monthly mean ambient temperatures across all U.S. states and Washington, D.C., from 1999 to 2020. The vertical red line indicates the reference temperature of 52.8 °F (11.6 °C), which corresponds to the overall mean temperature across all state-months and was used as the centering point for the main analysis.

The data of 9,108,644 ischemic heart disease deaths from 1999 to 2020 across all U.S. states were analyzed. The monthly ambient temperatures during this period across all states ranged from -12.8 °F (-24.9 °C) to 89.2 °F (31.8 °C), with a mean temperature of 52.8 °F (11.6 °C). The mean IHD mortality rate was 133.34 deaths per 100,000, with a range of 29.99 to 380.60 per 100,000 across states and years (Fig 1). The detailed number of deaths in the subgroups are given in the Table A in S1 Appendix.

**Fig 2:**
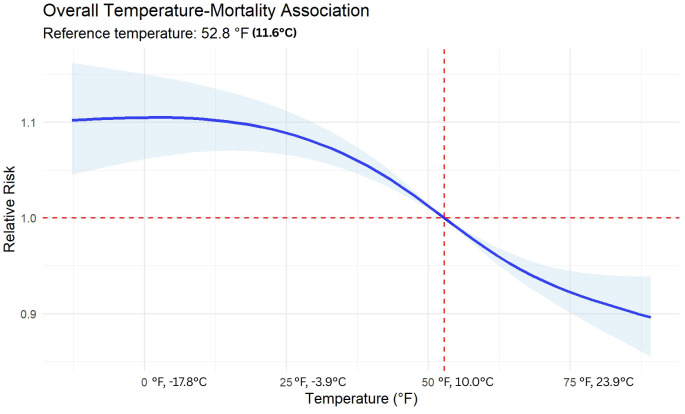
Overall temperature–mortality association for ischemic heart disease. The exposure–response curve illustrates the association between monthly mean ambient temperature and IHD mortality risk from 1999 to 2020. Relative risks (RRs) with 95% confidence intervals (shaded area) demonstrate a monotonic inverse relationship. The reference temperature (52.8 °F, 11.6 °C) is indicated by the vertical dashed line.

The analysis revealed a strong inverse association between ambient temperature and IHD mortality risk. The Wald test for the overall effect of the temperature was significant (Wald statistic = 210.03, p < 0.001), with significant point estimates observed at both extremes of the ambient temperature range. The risk of IHD mortality was highest at the lowest ambient temperature observed (-12.8 °F [-24.9 °C], RR 1.10, 95% CI: 1.05 - 1.16), corresponding to a 10% increase in risk compared with the reference. As ambient temperatures increased, the risk of mortality progressively declined. The minimum mortality temperature (MMT) corresponded to the highest ambient temperature observed (89.2 °F [31.8 °C], RR 0.90, 95% CI: 0.86 - 0.94), indicating a 10% decrease in the risk of mortality compared with the reference temperature. No increased risk of mortality was observed at higher ambient temperatures. Instead, the inverse association between IHD mortality risk and monthly mean ambient temperature persisted across the temperature range (Fig 2) (Table 1).

**Table 1:** Relative risks for ischemic heart disease in the overall population at select temperature points.

Temperature °F(°C)	Percentile	Relative Risk (95% CI)
-12.8 °F (-24.9 °C)	HMT (Cold)	1.10 (1.05 - 1.16)
22.5 °F (-5.3 °C)	5th (Cold)	1.09 (1.07 - 1.12)
39.3 °F (4.1 °C)	25^th^ (Cold)	1.05 (1.04 - 1.07)
52.8 °F (11.6 °C)	Mean	1.00 (Reference)
54.3 °F (12.4 °C)	50th	0.99 (0.99 - 0.99)
67.5 °F (19.7 °C)	75^th^ (Heat)	0.94 (0.93 - 0.96)
78.6 °F (25.9 °C)	95th (Heat)	0.92 (0.89 - 0.94)
89.2 °F (31.8 °C)	MMT (Heat)	0.90 (0.86 - 0.94)

Abbreviations: MMT: Minimum mortality temperature; HMT: Highest mortality temperature

### 3.2. Lagged effects

**Fig 3:**
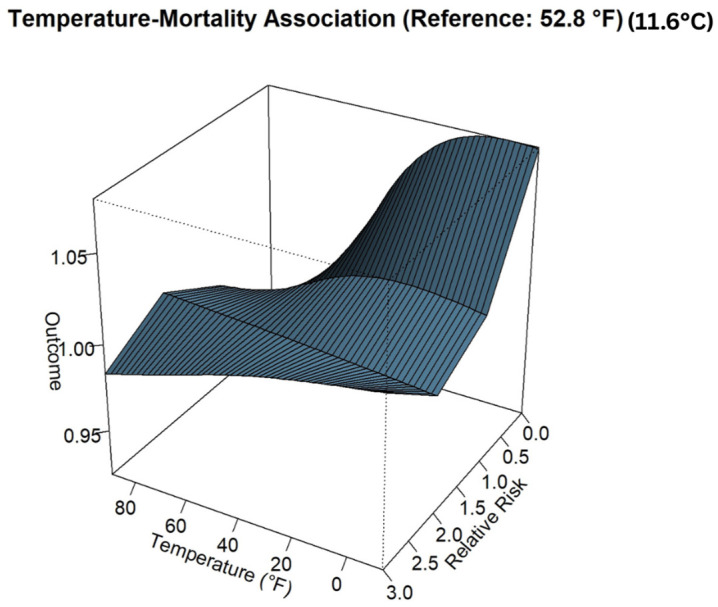
Three-dimensional Exposure-Lag-Response Surface of Temperature and IHD Mortality. This surface plot illustrates the non-linear relationship between monthly mean ambient temperature, time lags, and the relative risk (RR) of ischemic heart disease mortality, centered at 52.8°F (11.6°C). The model reveals a prominent, sustained increase in mortality risk at colder temperatures that persists across a 3-month lag period. Conversely, higher temperatures were associated with lower relative risk of mortality, with the lowest risk observed during the month of exposure.

**Fig 4:**
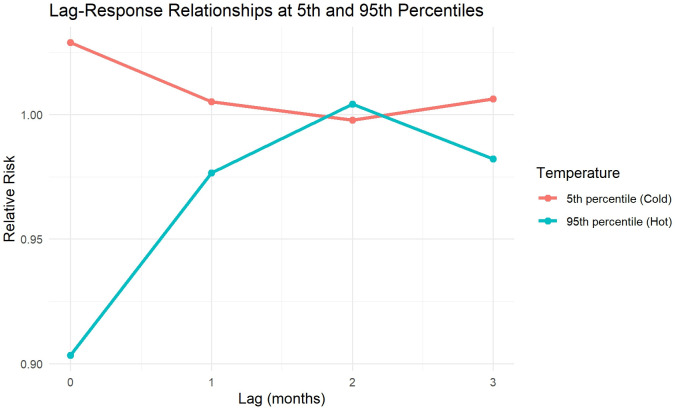
Lag-response relationships at the 5th and 95th percentile temperatures. The lag-response curves illustrate the temporal pattern of temperature-associated IHD mortality risk over a three-month lag period. The red line represents the 5th percentile temperature (22.5 °F, –5.3 °C), showing the highest risk at lag 0 with attenuation thereafter. The blue line represents the 95th percentile temperature (78.6 °F, 25.9 °C), demonstrating a similar decay pattern.

**Fig 5:**
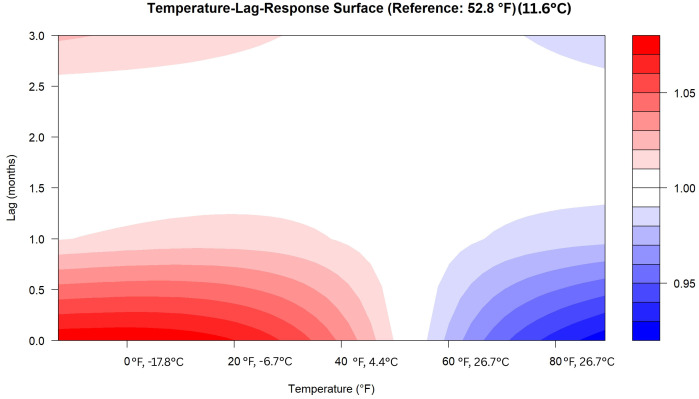
Contour Plot of the Temperature–Lag–Response Surface for IHD Mortality. This contour plot visualizes the interaction between monthly mean ambient temperature and time lags (0–3 months) on the relative risk (RR) of IHD mortality, using 52.8°F (11.6°C) as the reference. The intense red regions highlight a significant and immediate elevation in mortality risk at colder temperatures, which persists for approximately one month. Conversely, the blue regions indicate lower relative risk at higher temperatures, primarily concentrated during the month of initial exposure.

The lagged effects of temperature were also evaluated. The analysis revealed that the elevated mortality risk associated with cold exposure was most pronounced during the same month of exposure (lag 0) and gradually diminished in the subsequent months. Similarly, the inverse association at high ambient temperatures was strongest at lag 0 and weakened over time. These findings suggest that the mean ambient temperature of the corresponding month had the strongest association with IHD mortality, and the strength of this association for both high and low temperatures attenuated over time (Fig 3–5) (Table 2).

**Table 2:** Lagged effects of temperature on ischemic heart disease mortality in the overall population at the 5^th^ and 95^th^ percentile temperatures.

Temperature °F (°C)	Percentile	Lag (Months)	Relative Risk (95% CI)
22.5 °F (-5.3 °C)	5^th^ (Cold)	0	1.03 (1.02 - 1.04)
22.5 °F (-5.3 °C)		1	1.01 (1.00 - 1.01)
22.5 °F (-5.3 °C)		2	1.00 (0.99 - 1.00)
22.5 °F (-5.3 °C)		3	1.01 (1.00 - 1.01)
78.6 °F (25.9 °C)	95^th^ (Heat)	0	0.90 (0.89 - 0.92)
78.6 °F (25.9 °C)		1	0.98 (0.96 - 0.99)
78.6 °F (25.9 °C)		2	1.00 (0.99 - 1.02)
78.6 °F (25.9 °C)		3	0.98 (0.97 - 1.00)

### 3.3. Model fit and robustness

The model demonstrated a substantial improvement in fit, as evidenced by the reduction in deviance from the null model (235,722.17) to the fitted model (15,078.11). This improvement reflects the combined contribution of all included model components, state, year, and month fixed effects, long-term temporal trends, and the temperature cross-basis function, rather than the effect of any single variable. The Akaike Information Criterion (AIC = 132,690.29) further indicated satisfactory model performance. The dispersion parameter (θ = 333.80) suggested minimal overdispersion, confirming that the negative binomial specification was appropriate for the mortality data. Full model fit statistics are provided in Table B in S1 Appendix.

### 3.4. Subgroup analysis

(Fig A-E in S1 Appendix) (Table 3)

**Table 3:** Subgroup analyses of the effects of temperature on ischemic heart disease mortality at the 5^th^ and 95^th^ percentile temperature.

Subgroup	Temperature °F (°C)	Percentile	Relative Risk (95% CI)
**By Age**			
**Age < 45 years**	22.8 °F (-5.1°C)	5^th^ (Cold)	0.91 (0.59 - 1.40)
Wald statistic = 5.96, p = 0.74	8.4 °F (-13.1 °C)	95^th^ (Heat)	1.15 (0.72 - 1.83)
**Age 45–64 years**	22.6 °F (-5.2 °C)	5^th^ (Cold)	1.18 (1.12 - 1.24)
Wald statistic = 78.28, p < 0.001	78.3 °F (25.7 °C)	95^th^ (Heat)	0.92 (0.87 - 0.97)
**Age ≥ 65 years**	22.2 °F (-5.4 °C)	5^th^ (Cold)	1.12 (1.09 - 1.16)
Wald statistic = 141.49, p < 0.001	78.3 °F (25.7 °C)	95^th^ (Heat)	0.89 (0.86 - 0.93)
**By Sex**			
**Male**	22.5 °F (-5.3 °C)	5^th^ (Cold)	1.10 (1.07 - 1.13)
Wald statistic = 175.66, p < 0.001	78.6 °F (25.9 °C)	95^th^ (Heat)	0.91 (0.88 - 0.93)
**Female**	22.8 °F (-5.1 °C)	5^th^ (Cold)	1.09 (1.06 - 1.12)
Wald statistic = 156.12, p < 0.001	78.6 °F (25.9 °C)	95^th^ (Heat)	0.93 (0.89 - 0.96)
**By Race**			
**White**	22.5 °F (-5.3 °C)	5^th^ (Cold)	1.10 (1.08 - 1.13)
Wald statistic = 219.72, p < 0.001	78.6 °F (25.9 °C)	95^th^ (Heat)	0.91 (0.88 - 0.94)
**Black or African American**	28.2 °F (-2.1 °C)	5^th^ (Cold)	1.12 (1.07 - 1.18)
Wald statistic = 49.95, p < 0.001	80.2 °F (26.8 °C)	95^th^ (Heat)	0.91 (0.85 - 0.97)
**Asian or Pacific Islander**	28.5 °F (-1.9 °C)	5^th^ (Cold)	1.09 (1.02 - 1.17)
Wald statistic = 20.903, p = 0.013	80.5 °F (26.9 °C)	95^th^ (Heat)	1.01 (0.92 - 1.10)
**American Indian or Alaska Native**	32.3 °F (0.2 °C)	5^th^ (Cold)	1.01 (0.87 - 1.18)
Wald statistic = 5.78, p = 0.76	81.0 °F (27.2 °C)	95^th^ (Heat)	1.13 (0.90 - 1.43)
**By Climatic Region**			
**Cooler states**	17.3 °F (-8.2 °C)	5^th^ (Cold)	1.08 (1.05 - 1.13)
Wald statistic = 97.08, p < 0.001	71.7 °F (22.1 °C)	95^th^ (Heat)	0.86 (0.82 - 0.91)
**Warmer states**	34.5 °F (1.4 °C)	5^th^ (Cold)	1.07 (1.04 - 1.10)
Wald statistic = 92.29, p < 0.001	80.9 °F (27.2 °C)	95^th^ (Heat)	0.94 (0.91 - 0.98)

#### 3.4.1. By age.

The temperature-mortality association was statistically significant and consistent with the overall population in both the 45–64 years and ≥65 years subgroups. In both groups, lower ambient temperatures were associated with an increased risk of IHD mortality, while progressively warmer ambient temperatures were associated with decreased risk. The model performance was strong in both subgroups, as dispersion parameter (θ) suggested minimal overdispersion.

However, in individuals aged <45 years, no significant association was observed at any point across the temperature range. This subgroup demonstrated an extremely low dispersion parameter (θ = 1.19), indicating greater variability in the effect compared with the overall model. This variability may be related to the smaller and more heterogeneous event counts observed in this population. The absence of a statistically significant association in this subgroup should be interpreted cautiously and is likely attributable to limited statistical power rather than a confirmed absence of association. Raw death counts for this subgroup are provided in Supplementary Table 1.

#### 3.4.2. By sex.

In both male and female subgroups, the model revealed a statistically significant association between monthly mean ambient temperature and IHD mortality. The magnitude and direction of the effect was consistent with the pattern observed in the overall model, with no meaningful differences in either group. Furthermore, the dispersion parameter (θ) indicated minimal overdispersion in both groups, supporting the robustness and stability of the model.

#### 3.4.3. By race.

Among both White and Black or African American populations, the temperature-mortality association was statistically significant and closely aligned with the overall population. The model was stable in both subgroups, as dispersion parameter suggested minimal overdispersion.

In Asian or Pacific Islander individuals, the association was consistent with the overall pattern in the colder temperature range. However, the relative risk at the 95th percentile temperature was 1.01 (95% CI: 0.92–1.10), which was not statistically significant and the confidence interval crossed the null. This finding likely indicates uncertainty around the higher-temperature association in this subgroup rather than a confirmed absence of effect. Although the dispersion parameter indicated minimal overdispersion, the wider confidence intervals in this subgroup likely reflect smaller sample size and event counts, and estimates should be interpreted with caution.

Among American Indian or Alaska Native (Indigenous) populations, the temperature-mortality association was not statistically significant, with wide confidence intervals spanning the null at all temperature points. The extremely large dispersion parameter (θ = 8.68 × 10¹³), and the wide confidence intervals likely reflect both the small population size and limited events count, which reduced the precision of the estimates. This finding should be interpreted as inconclusive due to model instability and data sparsity, and not as evidence of no association. Raw death counts for this subgroup are provided in Supplementary Table 1.

#### 3.4.4. By climate.

Twenty-six states with a mean ambient temperature of 46.1°F (7.8 °C) were included in the cooler subgroup, while the warmer subgroup comprised D.C. and 24 states with a mean ambient temperature of 59.8°F (15.4 °C). Both groups demonstrated a statistically significant temperature-mortality association. The risk of IHD mortality was higher at lower ambient temperatures, and it decreased with a progressive increase in ambient temperature in both subgroups. Moreover, the model was stable in both groups, with a dispersion parameter (θ) indicating minimal overdispersion. Notably, in the cooler subgroup, the confidence interval for relative risk crossed the null value at very low ambient temperatures, although the direction of the effect remained consistent. This finding likely reflects the limited number of observations at very low ambient temperatures, which reduced the overall statistical power.

## 4. Discussion

### 4.1. Key findings

This nationwide ecological analysis of IHD mortality in the U.S. from 1999–2020 revealed a significant association between monthly mean ambient temperatures and IHD mortality. Lower temperatures were associated with higher IHD mortality, while warmer temperatures were associated with a lower mortality risk at the population level. At the coldest observed temperature (-12.8 °F, -24.9 °C), the relative risk (RR) was 1.10, indicating a 10% increase in mortality risk, whereas the warmest temperature (89.2 °F, 31.8 °C) corresponded to the minimum mortality temperature (RR 0.90), reflecting a 10% decrease. Overall, this indicates a 20% difference in IHD mortality risk across the temperature spectrum.

Lag analysis showed that the associations at both cold and warm temperatures were strongest in the month of exposure and diminished in subsequent months. The model demonstrated a substantial improvement in fit relative to the null model, reflecting the combined contribution of all model components including fixed effects and temporal adjustments. The dispersion parameter (θ = 333.80) suggested minimal overdispersion, confirming the appropriateness of the negative binomial specification and supporting the robustness of the observed associations.

The temperature–mortality relationship observed in this study differs from the commonly reported U- or J-shaped patterns in the literature, with a monotonic inverse association and no apparent increase in risk at higher temperatures [6–13]. This divergence can be explained by both methodological and population-level factors.

First, the temporal resolution of the exposure metric is likely a key contributor. Prior studies reporting U- or J-shaped relationships have predominantly used daily or sub-daily temperature data, which capture acute physiological responses to short-term heat extremes, including dehydration, arrhythmias, and increased cardiac workload. In contrast, the use of monthly mean temperature in the present analysis smooths over these short-term fluctuations. As a result, individual heatwave days with elevated risk may be diluted within the broader monthly average, attenuating the apparent effect of high temperatures. Therefore, the absence of a heat-related increase in mortality at the monthly level should not be interpreted as evidence that extreme heat does not pose cardiovascular risk, but rather reflects the temporal aggregation of exposure [22].Second, the observed pattern may also reflect population-level adaptation to heat in the United States. Widespread access to air conditioning, behavioral modifications, and public health interventions may mitigate the impact of sustained high temperatures on cardiovascular mortality [17]. In contrast, cold exposure is associated with physiological responses such as increased blood pressure, vasoconstriction, and heightened susceptibility to respiratory infections, including influenza, which may accumulate over longer periods and remain detectable at the monthly scale [23,24].

These findings are consistent with prior literature. For example, Barnett et al. (2007) reported declining heat-related cardiovascular mortality among U.S. elderly populations, while cold-related mortality persisted [17]. Similarly, Gasparrini et al. (2015), in a large multi-country study of over 74 million deaths, found that the majority of temperature-related mortality was attributable to moderately non-optimal temperatures, with a substantially greater contribution from cold than heat [25]. More recent evidence has also suggested lower IHD mortality at higher temperatures across diverse settings [18]. Importantly, these results should be interpreted as reflecting associations at the monthly population level and do not preclude the existence of short-term increases in IHD risk during extreme heat events.

A lag period of up to three months was examined to capture potential delayed effects of ambient temperature on IHD mortality. However, lag-specific analyses indicated that the association was strongest within the first month (lag 0), with no statistically significant effects observed beyond this period. Accordingly, the primary association appears to be driven by same-month exposure, and the extended lag window was included to ensure that any potential delayed effects were adequately assessed.

Subgroup analysis revealed notable heterogeneity. Adults under 45 showed no significant temperature-mortality association, with considerable model instability likely due to fewer events. However, the absence of statistical significance in this group should not be interpreted as a confirmed null effect, as reduced statistical power from low event counts limits inference. In contrast, adults ≥45 exhibited associations consistent with the overall trend, with stable exposure-response curves. The effect was also consistent across sex, with males and females showing comparable results.

Race-specific analysis showed that White and Black populations experienced associations consistent with the overall population pattern, with elevated mortality risk at lower temperatures and reduced risk at higher temperatures. Among Asian or Pacific Islander populations, the association persisted at colder temperatures, but the estimate at higher temperatures was imprecise, with a wide confidence interval crossing the null, indicating uncertainty rather than a confirmed absence of effect. These findings should be interpreted cautiously given the smaller sample size and event counts in this subgroup. Indigenous populations showed no significant associations, with extremely large dispersion (θ = 8.68 × 10¹³) and wide confidence intervals, indicating model instability due to data sparsity and limiting conclusions. These findings highlight the need for further study in Asian and Indigenous groups.

To further assess whether the associations differed by geography, climate and acclimatization, we divided the states into cooler and warmer groups. In both groups, the temperature–mortality association was consistent and in line with the overall trend. Notably, cooler states showed a stronger inverse association at higher ambient temperatures (RR 0.86) compared with the warmer states (RR 0.94), while the association at cold temperatures was comparable between groups.

### 4.2. Public health and policy implications

Our findings identify a significant inverse association between ambient temperature and IHD mortality, with lower temperatures associated with higher mortality risk at the population level. Between extremes of ambient temperature, we observed a net 20% difference in mortality risk. On a population level, even modest percentage changes can translate into substantial numbers of excess deaths, given the high burden of IHD. This emphasizes the need for targeted winter interventions such as influenza vaccination, improved heating access, and community cold-weather preparedness. Preventive measures should particularly focus on high-risk populations, including the elderly and those with pre-existing cardiovascular conditions.

The absence of excess IHD mortality in warmer months is consistent with evidence of better population-level adaptation to heat in the U.S., including widespread air conditioning and behavioral modification, though it does not exclude the possibility of acute heat-related cardiovascular risk [17]. Heat-action plans and preparedness strategies for extreme heat events should not be deprioritized on the basis of these findings. The independent role of short-term extremes such as heat waves cannot be excluded and warrants specific preparedness measures [22].

Given the consistent associations across sex, age ≥ 45, and both White and Black populations, broad public health strategies can be employed nationwide. However, subgroup differences, particularly among Asian and Indigenous populations, highlight the need for further research to understand potential biological, cultural, or environmental modifiers of risk.

### 4.3. Strengths and limitations

This study has several strengths. It used over two decades of publicly available nationwide ambient temperature and mortality data, covering all U.S. states and ensuring reproducibility. Potential coding bias was minimized by using standardized national mortality data. The large dataset provided high statistical power and reduced the risk of random error. The use of negative binomial regression within a distributed lag non-linear model (DLNM) captured both non-linear and delayed effects of ambient temperature while minimizing overdispersion. The model demonstrated a substantial improvement in fit relative to the null model, reflecting the combined contribution of all included model components. Fixed effects for year, month, and state helped control potential confounding. Finally, subgroup analyses by age, sex, race, and climate explored potential heterogeneity, while sensitivity analyses confirmed the robustness of the findings.

This study has several limitations.

1. This study is ecological in design and utilizes aggregated state-level data, which precludes the assessment of individual-level exposure, outcomes, and covariates. As a result, the findings do not account for within-state heterogeneity in ambient temperature or individual cardiovascular risk factors. This introduces the possibility of ecological fallacy, whereby associations observed at the population level may not accurately reflect relationships at the individual level. Therefore, the observed associations between ambient temperature and IHD mortality should be interpreted as ecological associations and not as causal effects at the individual level. While these findings may generate hypotheses regarding potential temperature-related cardiovascular risks, causal inference cannot be established from this study's design.
2. Although we adjusted for state, year, and month fixed effects and long-term temporal trends, residual confounding remains a possibility. In particular, we were unable to directly adjust for important time-varying factors such as air pollution (e.g., PM2.5 and ozone), influenza activity, socioeconomic changes, heating practices, and healthcare access. These factors may be correlated with ambient temperature and could partially explain the observed association between colder temperatures and higher IHD mortality. Therefore, our findings should be interpreted as associative rather than causal, and the potential influence of unmeasured confounding cannot be excluded.
3. This study used state-level temperature and mortality data, which may introduce exposure misclassification due to substantial within-state climate heterogeneity. Such non-differential measurement error may attenuate observed associations, potentially biasing effect estimates toward the null. Consequently, localized temperature–mortality relationships may be underestimated in this analysis.
4. The use of monthly mean ambient temperatures is a substantive limitation. Monthly aggregation smooths over daily temperature fluctuations and cannot capture the acute physiological effects of short-duration heat extremes such as heatwaves. The absence of heat-related excess IHD mortality at monthly resolution should therefore not be interpreted as evidence that extreme heat poses no cardiovascular risk. Rather, it suggests that over sustained monthly periods, population-level adaptations, including widespread air conditioning, behavioral modification, and acclimatization, may reduce the net monthly mortality signal attributable to heat in the United States. Studies using daily or hourly resolution are better suited to characterizing acute temperature extremes and heatwave-specific cardiovascular risk.
5. Several subgroups, particularly Asian or Pacific Islander and American Indian or Alaska Native populations, had relatively small sample sizes and event counts, leading to imprecise and in some cases unstable estimates. The American Indian or Alaska Native subgroup exhibited substantial model instability, as indicated by the extremely large dispersion parameter (θ = 8.68 × 10¹³). Accordingly, the null finding in this subgroup should not be interpreted as evidence of no association but rather as an inconclusive result due to data sparsity.
6. The classification of states into “cooler” and “warmer” groups was based on an operational threshold of 52.8 °F (11.6 °C), corresponding to the mean temperature used in the main analysis. We acknowledge that this is not a standard climatological cutoff and may not fully capture geographic or climate heterogeneity. Therefore, these subgroup findings should be interpreted with caution, and future studies should consider established climate classification systems such as Köppen–Geiger climate zones or NOAA climate regions.
7. While CDC WONDER uses standardized coding validated by the National Vital Statistics System, some degree of misclassification of underlying cause of death is possible, which may introduce non-differential outcome misclassification and potentially attenuate observed associations.
8. The findings may not be generalizable beyond the U.S. context.

## 5. Conclusions

This nationwide ecological analysis spanning 22 years demonstrates a significant inverse association between monthly mean ambient temperature and ischemic heart disease mortality in the United States. Lower monthly mean ambient temperatures were associated with up to a 20% higher relative risk of IHD mortality compared with the warmest observed temperatures, with the strongest associations observed during the month of exposure. These findings remained consistent across most demographic subgroups, though associations were not significant in adults under 45 years and some racial minority populations, likely reflecting limited statistical power in smaller subgroups rather than confirmed null effects.

These findings identify colder ambient temperatures as a significant ecological correlate of IHD mortality in the United States and underscore the potential value of targeted public health interventions during winter months. Winter preparedness strategies, including influenza vaccination campaigns, improved heating access for vulnerable populations, and cold-weather health advisories, should be prioritized to reduce cold-associated cardiovascular risk. While heat-related risks appear less prominent at the population level in this monthly analysis, continued monitoring and preparedness for extreme heat events remain important components of comprehensive climate-health adaptation policies. These findings should be interpreted as ecological associations and not as evidence of individual-level causal effects. Future studies incorporating individual-level data, direct confounding adjustment, and daily temperature resolution are needed to further characterize the nature and magnitude of this relationship.

## Supporting information

S1 Appendix This appendix contains Table A, Table B, and Figs A–E. **Table A. Total ischemic heart disease mortality counts and population at risk by demographic subgroup, United States, 1999–2020.** The table presents cumulative IHD deaths and person-years (in thousands) across subgroups. Overall, 9.1 million IHD deaths occurred among 6.7 billion person-years. The largest burden was in adults ≥65 years (7.4 million deaths) and White populations (7.9 million deaths), reflecting population distribution. **Table B. Model fit and robustness indicators for subgroup analyses.** The table shows null deviance, residual deviance, AIC, and dispersion parameter (θ) for each subgroup. θ refers to the overdispersion parameter in the NB2 parameterization. The extremely large θ for Indigenous populations (8.68 × 10¹³) indicates model instability due to small sample size, precluding valid inference for this subgroup. **Fig A. Subgroup analysis by age.** The top-left panel shows no significant association in individuals <45 years. The top-right panel shows a significant association consistent with the overall effect in those aged 45–64 years. The bottom-left panel demonstrates a significant association in individuals ≥65 years, reflecting heightened vulnerability in older populations. **Fig B. Subgroup analysis by sex.** The left panel shows a significant association in males, while the right panel shows a significant association in females, both consistent with the overall population-level effect. **Fig C. Subgroup analysis by race**The top-left panel shows a significant association in White populations consistent with the overall trend. The top-right panel demonstrates a similar pattern in Black populations. The bottom-left panel indicates that Asian populations show a significant effect at cold extremes but not at hot extremes, while the bottom-right panel shows no significant association in Indigenous populations. Both Asian and Indigenous subgroups exhibit wide confidence intervals, likely reflecting smaller sample sizes. **Fig D. Subgroup analysis by climate.** The left panel shows significant and consistent associations in cooler regions, with wider confidence intervals at extreme low temperatures, likely reflecting smaller populations and fewer events. The right panel shows significant and consistent associations in warmer regions, both following the overall trend observed in the full population. **Fig E. Classification of U.S. states by climatic region.** Average monthly mean temperatures from 1999–2020 were calculated for each state. States below the national mean (52.8 °F) were classified as cooler, while those above were classified as warmer, forming two distinct climatic groups for subgroup analysis.
(DOCX)

## Abbreviations

IHD: ischemic heart disease
CDC: centers for disease control and prevention
ICD-10: international classification of diseases, 10th revision
NCEI: National centers for environmental information
RR: relative risk
CI: confidence interval
DLNM: distributed lag non-linear model
AIC: Akaike information criterion
df: degrees of freedom
MMT: minimum mortality temperature
