# Supplementary material for: The association between monthly ambient temperatures and ischemic heart disease mortality in the United States: A nationwide ecological analysis, 1999–2020
Source: PLOS Glob Public Health. 2026 Jun 18;6(6):e0006641. doi: 10.1371/journal.pgph.0006641 (PMC13278391; doi:10.1371/journal.pgph.0006641)
Supplement: S1 Appendix — Table A. Total ischemic heart disease mortality counts and population at risk by demographic subgroup, United States, 1999–2020. The table presents cumulative IHD deaths and person-years (in thousands) across subgroups. Overall, 9.1 million IHD deaths occurred among 6.7 billion person-years. The largest burden was in adults ≥65 years (7.4 million deaths) and White populations (7.9 million deaths), reflecting population distribution. Table B. Model fit and robustness indicators for subgroup analyses. The table shows null deviance, residual deviance, AIC, and dispersion parameter (θ) for each subgroup. θ refers to the overdispersion parameter in the NB2 parameterization. The extremely large θ for Indigenous populations (8.68 × 10¹³) indicates model instability due to small sample size, precluding valid inference for this subgroup. Fig A. Subgroup analysis by age. The top-left panel shows no significant association in individuals <45 years. The top-right panel shows a significant association consistent with the overall effect in those aged 45–64 years. The bottom-left panel demonstrates a significant association in individuals ≥65 years, reflecting heightened vulnerability in older populations. Fig B. Subgroup analysis by sex. The left panel shows a significant association in males, while the right panel shows a significant association in females, both consistent with the overall population-level effect. Fig C. Subgroup analysis by raceThe top-left panel shows a significant association in White populations consistent with the overall trend. The top-right panel demonstrates a similar pattern in Black populations. The bottom-left panel indicates that Asian populations show a significant effect at cold extremes but not at hot extremes, while the bottom-right panel shows no significant association in Indigenous populations. Both Asian and Indigenous subgroups exhibit wide confidence intervals, likely reflecting smaller sample sizes. Fig D. Subgroup analysis by climate. The [file pgph.0006641.s001.docx]

**S1_Appendix**

This appendix contains Table A, Table B, and Figs A–E.

**Contents:**

1. Table A. Total ischemic heart disease mortality counts and population at risk by demographic subgroup, United States, 1999–2020
2. Table B. Model fit and robustness indicators for subgroup analyses
3. Fig A. Subgroup analysis by age
4. Fig B. Subgroup analysis by sex
5. Fig C. Subgroup analysis by race
6. Fig D. Subgroup analysis by climate
7. Fig E. Classification of U.S. states by climate

| **Subgroup** | **IHD Mortality** | **Total Population** |
| --- | --- | --- |
| **Overall** | 9,108,644 | 6,746,356,647 |
| **By Age** |  |  |
| < 45 Years | 156,272 | 4,123,878,915 |
| 45-64 Years | 1,547,869 | 1,694,001,067 |
| ≥ 65 Years | 7,403,902 | 928,476,665 |
| **By Sex** |  |  |
| Male | 4,939,611 | 3,317,352,843 |
| Female | 4,169,033 | 3,429,003,804 |
| **By Race** |  |  |
| White | 7,917,461 | 5,367,045,067 |
| Black or African American | 966,132 | 919,034,937 |
| Asian or Pacific Islander | 182,409 | 371,914,051 |
| American Indian or Alaska Native | 42,642 | 88,362,592 |

**Table A. Total ischemic heart disease mortality counts and population at risk by demographic subgroup, United States, 1999–2020**

The table presents cumulative IHD deaths and person-years (in thousands) across subgroups. Overall, 9.1 million IHD deaths occurred among 6.7 billion person-years. The largest burden was in adults ≥65 years (7.4 million deaths) and White populations (7.9 million deaths), reflecting population distribution.

| **Subgroup** | **Null Deviance** | **Residual Deviance** | **AIC** | **Theta (θ)** |
| --- | --- | --- | --- | --- |
| **Overall** | 235,722.17 | 15,078.11 | 132,690.29 | 333.80 |
| **Age** |  |  |  |  |
| <45 years | 16,044.7 | 7,949.05 | 39,879.8 | 1.19 |
| 45-64 years | 100,413.06 | 22,552.94 | 88,620.7 | 248.70 |
| ≥65 years | 199,302.28 | 12,689.74 | 95,832.76 | 339.71 |
| **Sex** |  |  |  |  |
| Male | 150,012.99 | 14,467.39 | 119,309.3 | 418.57 |
| Female | 235,530.88 | 14557.29 | 116,120.31 | 306.385 |
| **Race** |  |  |  |  |
| White | 233,688.12 | 14,703.87 | 128,703.66 | 350.33 |
| Black or African American | 59,586.88 | 8,202.52 | 64,474.23 | 219.18 |
| Asian or Pacific Islander | 18,374.94 | 2,270.85 | 17,992.98 | 191.53 |
| American Indian or Alaska Native | 5,961.14 | 618.53 | 6,024 | 8.68 × 10¹³ |
| **Climatic Region** |  |  |  |  |
| Cooler states | 115,871.25 | 7,620.93 | 65,120.9 | 272.88 |
| Warmer states | 123,334.08 | 7,353.47 | 67,683 | 430.69 |

**Table B. Model fit and robustness indicators for subgroup analyses**

The table shows null deviance, residual deviance, AIC, and dispersion parameter (θ) for each subgroup. θ refers to the overdispersion parameter in the NB2 parameterization. The extremely large θ for Indigenous populations (8.68 × 10¹³) indicates model instability due to small sample size, precluding valid inference for this subgroup.


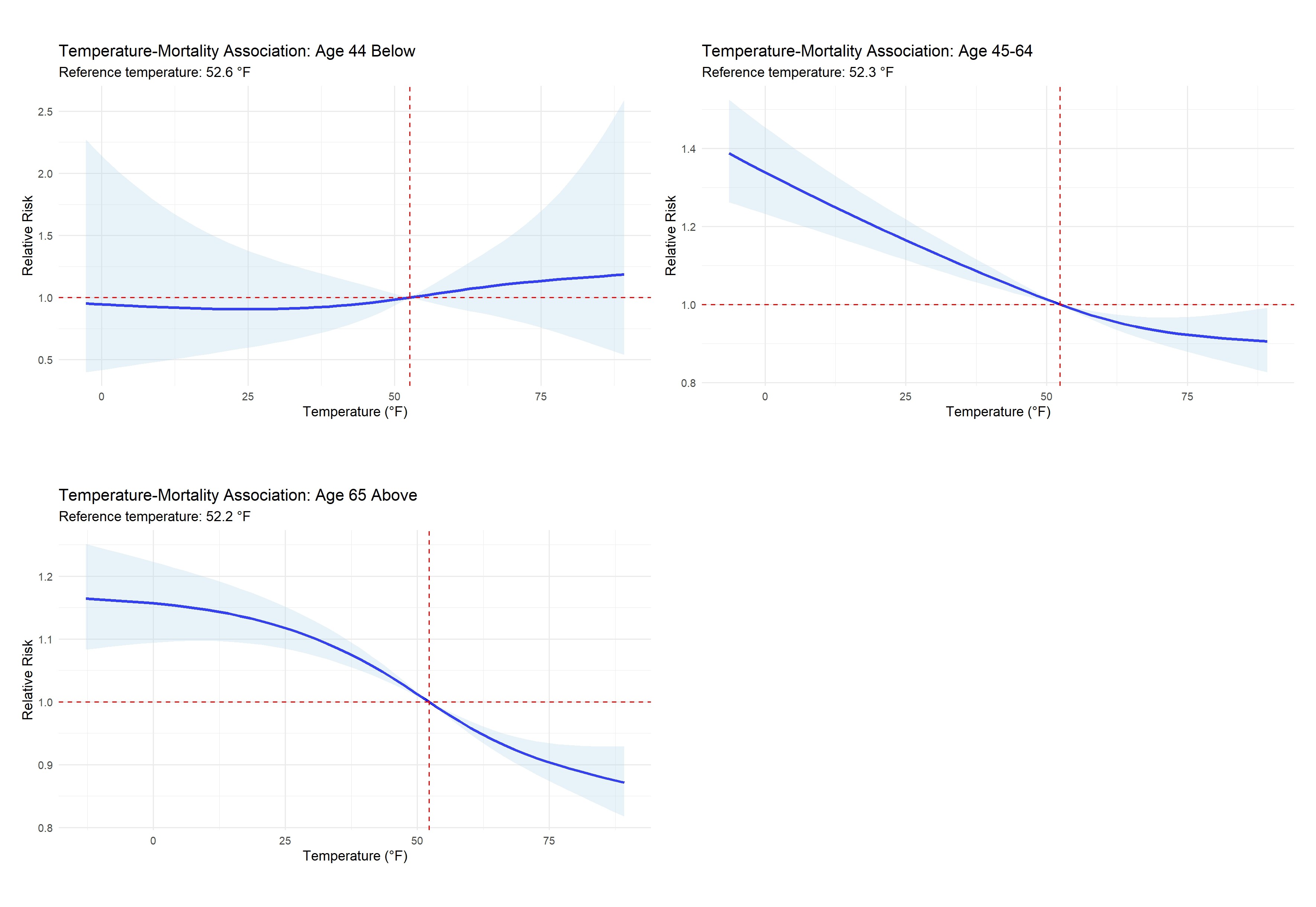
**Fig A. Subgroup analysis by age**
The top-left panel shows no significant association in individuals <45 years. The top-right panel shows a significant association consistent with the overall effect in those aged 45–64 years. The bottom-left panel demonstrates a significant association in individuals ≥65 years, reflecting heightened vulnerability in older populations.


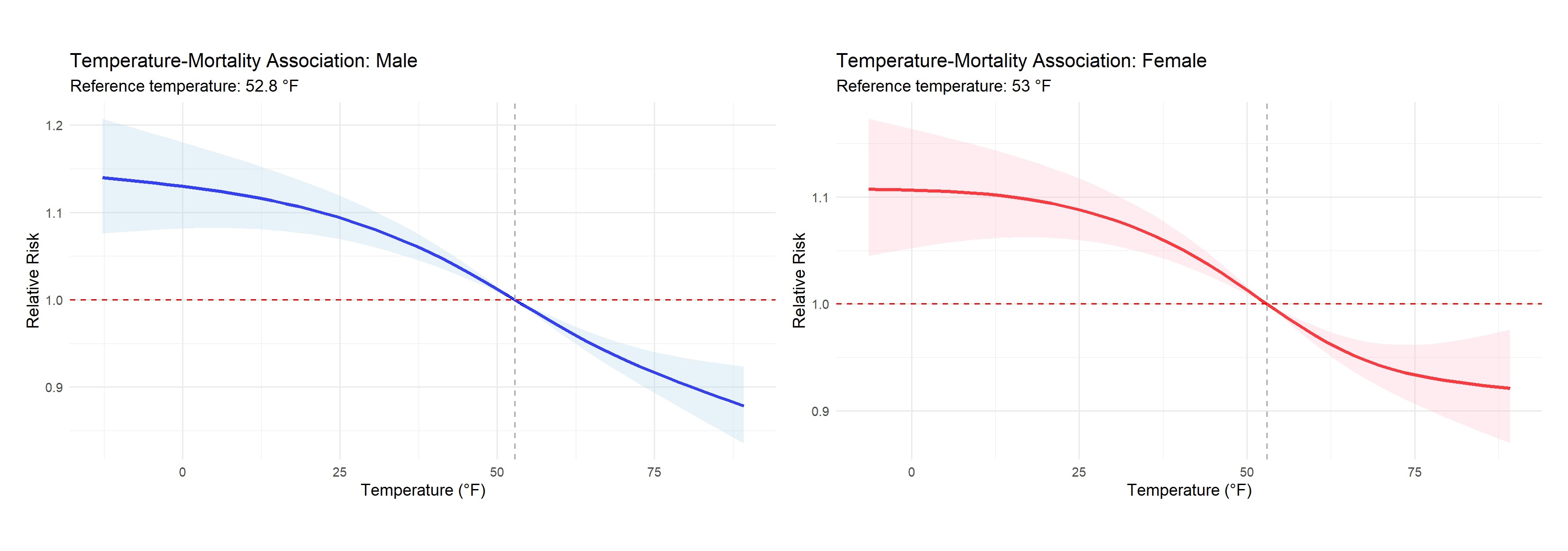
**Fig B. Subgroup analysis by sex**
The left panel shows a significant association in males, while the right panel shows a significant association in females, both consistent with the overall population-level effect.


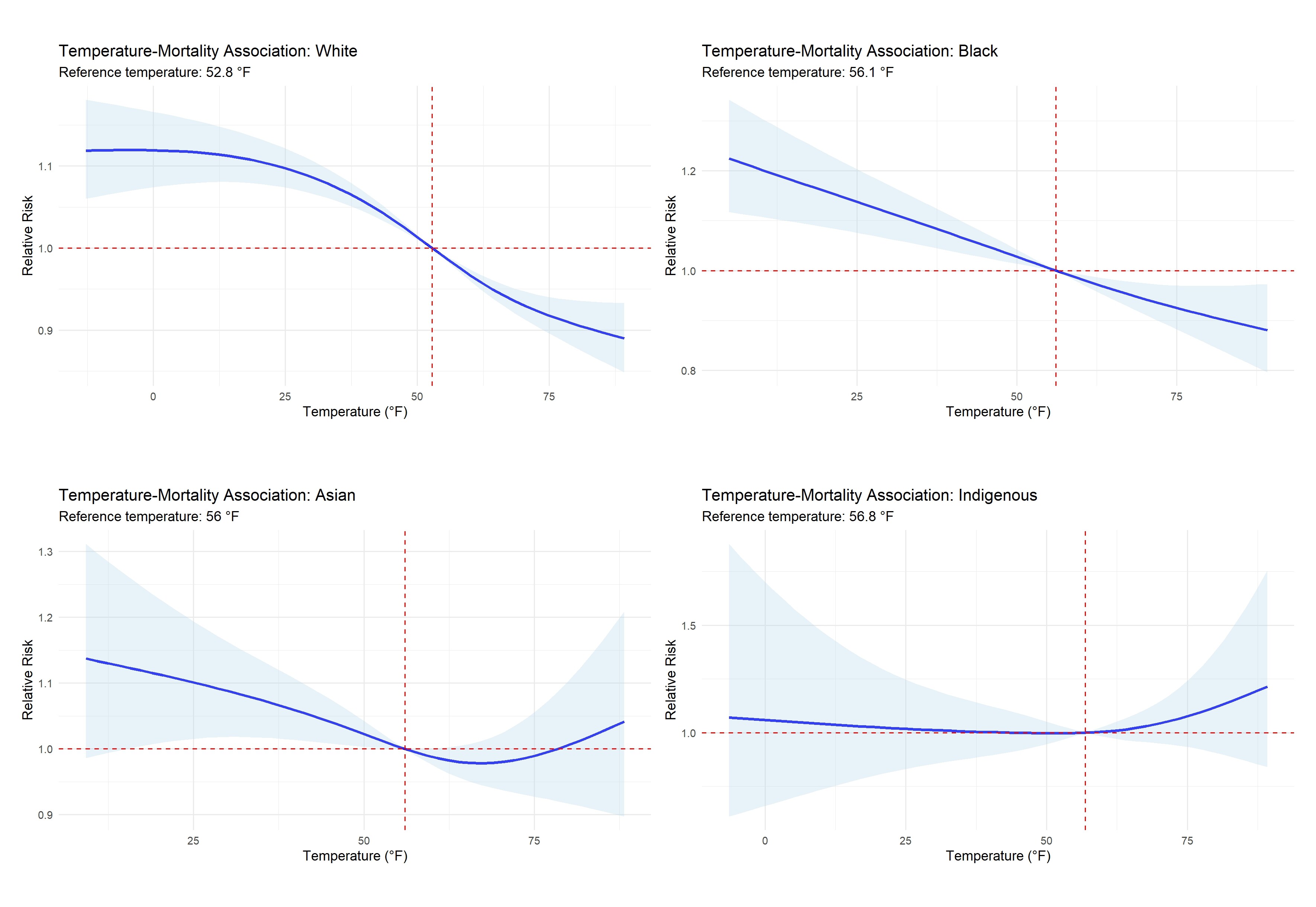
**Fig C. Subgroup analysis by race**
The top-left panel shows a significant association in White populations consistent with the overall trend. The top-right panel demonstrates a similar pattern in Black populations. The bottom-left panel indicates that Asian populations show a significant effect at cold extremes but not at hot extremes, while the bottom-right panel shows no significant association in Indigenous populations. Both Asian and Indigenous subgroups exhibit wide confidence intervals, likely reflecting smaller sample sizes.


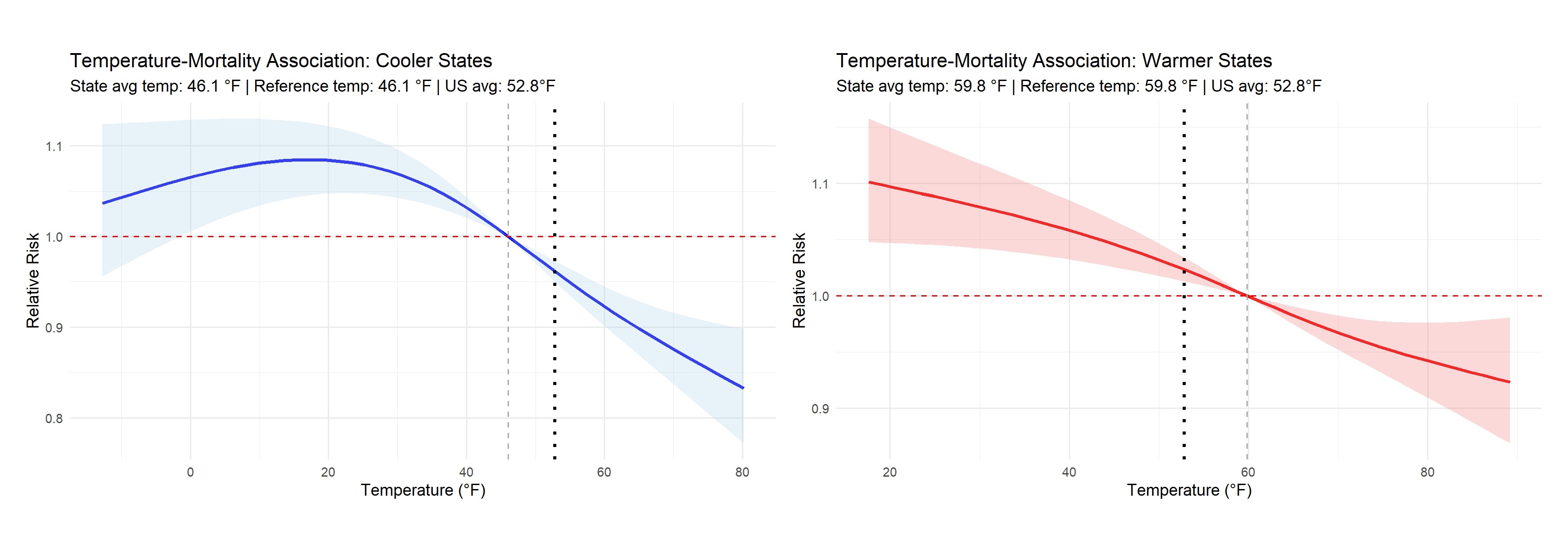
**Fig D. Subgroup analysis by climate**
The left panel shows significant and consistent associations in cooler regions, with wider confidence intervals at extreme low temperatures, likely reflecting smaller populations and fewer events. The right panel shows significant and consistent associations in warmer regions, both following the overall trend observed in the full population.


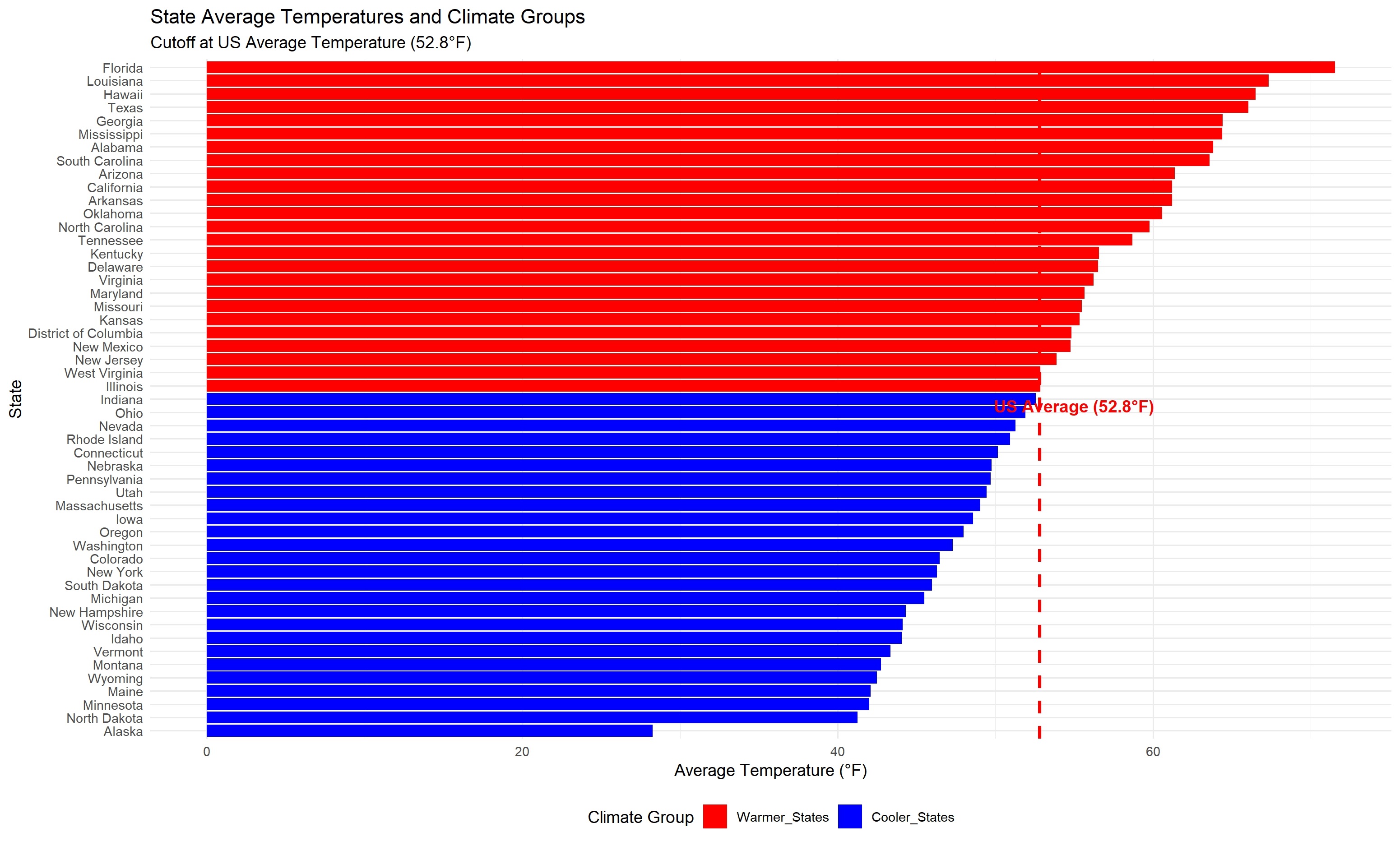
**Fig E. Classification of U.S. states by climate**
Average monthly mean temperatures from 1999–2020 were calculated for each state. States below the national mean (52.8 °F) were classified as cooler, while those above were classified as warmer, forming two distinct climatic groups for subgroup analysis.
