# Supplementary material for: The association between monthly ambient temperatures and ischemic heart disease mortality in the United States: A nationwide ecological analysis, 1999–2020
Source: PLOS Glob Public Health. 2026 Jun 18;6(6):e0006641. doi: 10.1371/journal.pgph.0006641 (PMC13278391; doi:10.1371/journal.pgph.0006641)
Supplement: Attachment — Submitted filename: Reviewers Report PONE-D-25-63906.pdf [file pgph.0006641.s002.pdf]

REVIEWER'S ANALYSIS

Manuscript Title: *The Population Burden of Ischemic Heart Disease Mortality Attributable to Ambient Temperature in the United States: A Nationwide Ecological Analysis, 1999–2020*

Article Type: Research Article

Reviewer Recommendation: Major Revision

1. INTRODUCTION

This manuscript presents a nationwide ecological time-series analysis examining associations between monthly mean ambient temperature and ischemic heart disease (IHD) mortality in the United States from 1999–2020. Using CDC WONDER mortality data and NOAA temperature records, the authors apply a negative binomial distributed lag non-linear model (DLNM) to quantify exposure–response relationships and subgroup heterogeneity.

The study addresses a topic of high public health relevance, climate-related cardiovascular mortality, and uses large-scale, publicly available datasets over a long temporal window. The modeling framework is generally appropriate for count data with overdispersion and delayed effects. However, several substantive conceptual and methodological issues must be addressed before the manuscript can be considered for publication, which will be described below.

2. SUMMARY OF THE STUDY

The authors analyzed monthly state-level IHD mortality (ICD-10 I20–I25) from 1999–2020 across all 50 U.S. states and Washington, D.C., using data from Centers for Disease Control and Prevention (CDC WONDER). Monthly mean state-level temperature data were obtained from National Centers for Environmental Information.

A negative binomial regression with DLNM was used to estimate non-linear and lagged associations (lag 0–3 months), adjusting for state, year, and month fixed effects and long-term trends. The authors report:

- Inverse exposure–response relationship.
- Increased mortality at low temperatures (RR 1.10 at −12.8°F).
- Protective association at higher temperatures (RR 0.90 at 89.2°F).
- No evidence of increased mortality at higher temperatures.
- Stronger associations in adults ≥45 years.
- Consistency across most subgroups.

They conclude that cold exposure is a substantial and preventable driver of IHD mortality in the U.S.

3. MAJOR COMMENTS

2.1 Attribution and "Population Burden" Language: The manuscript repeatedly refers to a "population burden" attributable to temperature. However, there´s no:

- attributable fraction (AF) or attributable number of deaths is estimated;
- counterfactual exposure scenario is defined;
- cumulative temperature-attributable mortality is computed.

Thus, the term "burden" without formal burden estimation (e.g., excess deaths relative to minimum mortality temperature), is overstated.

The required revision is to conduct a proper temperature-attributable mortality analysis (e.g., cumulative AF across distribution), or revise the title, abstract, and conclusions to remove attribution/burden terminology and instead describe associations.

2.2 Interpretation of Model Fit ("93.6% Variability Explained"): The statement that the model "explains 93.6% of spatiotemporal variability" is statistically problematic. Moreover, the large deviance reduction from null to fitted model largely reflects inclusion of state fixed effects, strong seasonal effects (month) and long-term time trends.

It cannot be interpreted as temperature explaining 93.6% of mortality variability. It is misleading to attribute that explanatory power to temperature. It´s important to reframe this statement to clarify that the overall model fit improved substantially, without attributing explanatory power specifically to temperature.

2.3 Ecological Design and Causal Language: This is a state-level ecological study using monthly aggregates and the manuscript uses strong causal language (e.g. "preventable environmental risk factor"). Therefore, no individual-level exposure is measured and no within-state microclimate variation is captured. Additionally, there is no adjustment for individual cardiovascular risk factors.

It is imperative to maintain a balanced and objective perspective in the use of causal language. It is imperative to accentuate associative findings and to engage in a thorough discussion regarding the potential for ecological fallacy.

2.4 Confounding: Insufficient Control: The models adjust for state, year, month, and long-term trends. However, important potential confounders are not addressed, such as:

- Air pollution (PM2.5, ozone)
- Influenza season severity
- Socioeconomic variation over time
- Heating fuel access
- Healthcare access

Given known winter correlations between cold temperature, influenza, and air pollution, the cold effect may be partially confounded. This limitation is acknowledged superficially but not critically evaluated.

The authors should expand the discussion to critically evaluate the potential magnitude and direction of residual confounding and how it may influence interpretation of the cold effect.

2.5 Exposure Metric: Monthly Mean Temperature: The use of monthly mean temperature substantially limits inference because masks short-term extremes, becomes acute heatwave effects indetectable and likely attenuates high-temperature risk or acute cardiovascular triggers.

The absence of a heat-related increase in mortality may reflect temporal aggregation bias rather than true absence of risk. Thus, the authors should discuss how monthly aggregation may attenuate extreme temperature effects and interpret the monotonic inverse association cautiously.

2.6 Climate Subgroup Classification: The classification of states into “cooler” and “warmer” groups using the overall mean temperature (52.8°F) as a threshold is arbitrary. This approach does not reflect established climatological classifications and mixes temporal variation with structural climate differences.

The authors should provide stronger justification for this threshold or consider alternative classification schemes (e.g., climatological zones or long-term annual means).

2.7 Racial Subgroup Instability: The American Indian/Alaska Native subgroup shows an extremely large dispersion parameter ($\theta \approx 8.68 \times 10^{13}$), suggesting model instability and the interpretation of null effects in this subgroup should be made cautiously.

The authors should clarify model stability issues and avoid overinterpreting non-significant findings in small subgroups.

4. MINOR COMMENTS

1. The abstract overstates novelty (“first study”) without strong substantiation.
2. The MMT coinciding with the highest observed temperature suggests model monotonicity; discuss biological plausibility.
3. Clarify whether temperature was entered in °F or standardized.
4. Provide number of total deaths analyzed.
5. Specify population offset source.
6. Clarify whether overdispersion parameter (θ) refers to NB2 parameterization.
7. Several references appear miscited or future-dated (e.g., 2025 NEJM citation).
8. Ensure adherence to STROBE reporting checklist (supplement should be provided).

5. RECOMMENDATION

Decision: Major Revision

The dataset and modeling approach are promising, and the study has potential to make a meaningful contribution. With substantial revision, this manuscript could be suitable for publication in PLOS Global Public Health. However, revisions are required to:

1. Correct overstated attribution language.
2. Clarify model interpretation.
3. Strengthen limitations and confounding discussion.
4. Reframe conclusions to avoid causal overreach.
5. Improve methodological transparency.
