# Supplementary material for: The association between monthly ambient temperatures and ischemic heart disease mortality in the United States: A nationwide ecological analysis, 1999–2020
Source: PLOS Glob Public Health. 2026 Jun 18;6(6):e0006641. doi: 10.1371/journal.pgph.0006641 (PMC13278391; doi:10.1371/journal.pgph.0006641)
Supplement: Peer review history

# Peer review: Decision Letter 0

**DOI:** 10.1371/journal.pgph.0006641.r001

**Authors:** Katrien Janin

24 Mar 2026

PGPH-D-26-00370

“The Population Burden of Ischemic Heart Disease Mortality Attributable to Ambient Temperature in the United States: A Nationwide Ecological Analysis, 1999–2020”

PLOS Global Public Health

Dear Dr. Ahmed,

Thank you for submitting your manuscript to PLOS Global Public Health. After careful consideration, we feel that it has merit but does not fully meet PLOS Global Public Health’s publication criteria as it currently stands. Therefore, we invite you to submit a revised version of the manuscript that addresses the points raised during the review process.

Your manuscript has been evaluated by two reviewers. While they expressed interest in your work, they also identified several concerns that need to be addressed. Please consider their comments carefully when preparing your revised manuscript and your point‑by‑point response.

Please submit your revised manuscript by May 08 2026 11:59PM. If you will need more time than this to complete your revisions, please reply to this message or contact the journal office at globalpubhealth@plos.org. When you're ready to submit your revision, log on to https://www.editorialmanager.com/pgph/ and select the 'Submissions Needing Revision' folder to locate your manuscript file.

Please include the following items when submitting your revised manuscript:

- A letter that responds to each point raised by the editor and reviewer(s). You should upload this letter as a separate file labeled 'Response to Reviewers'.
- A marked-up copy of your manuscript that highlights changes made to the original version. You should upload this as a separate file labeled 'Revised Manuscript with Track Changes'.
- An unmarked version of your revised paper without tracked changes. You should upload this as a separate file labeled 'Manuscript'.

Guidelines for resubmitting your figure files are available below the reviewer comments at the end of this letter.

As the corresponding author, your ORCID iD is verified in the submission system and will appear in the published article. PLOS supports the use of ORCID, and we encourage all coauthors to register for an ORCID iD and use it as well. Please encourage your coauthors to verify their ORCID iD within the submission system before final acceptance, as unverified ORCID iDs will not appear in the published article. *Only*  the individual author can complete the verification step; PLOS staff *cannot*  verify ORCID iDs on behalf of authors.

We look forward to receiving your revised manuscript.

Kind regards,

Katrien G. Janin, PhD

Staff Editor

PLOS Global Public Health

Journal Requirements:

1. Please provide separate figure files in .tif or .eps format.

For more information about figure files please see our guidelines:  LINK

https://journals.plos.org/globalpublichealth/s/figures

https://journals.plos.org/globalpublichealth/s/figures#loc-file-requirements

2. Some material included in your submission may be copyrighted. According to PLOS’s copyright policy, authors who use figures or other material (e.g., graphics, clipart, maps) from another author or copyright holder must demonstrate or obtain permission to publish this material under the Creative Commons Attribution 4.0 International (CC BY 4.0) License used by PLOS journals. Please closely review the details of PLOS’s copyright requirements here: PLOS Licenses and Copyright. If you need to request permissions from a copyright holder, you may use PLOS's Copyright Content Permission form.

Please respond directly to this email or email the journal office and provide any known details concerning your material's license terms and permissions required for reuse, even if you have not yet obtained copyright permissions or are unsure of your material's copyright compatibility.

Potential Copyright Issues:

"Graphical Abstract.png" : Please confirm whether you drew the images / clip-art within the figure panels by hand. If you did not draw the images, please provide (a) a link to the source of the images or icons and their license / terms of use; or (b) written permission from the copyright holder to publish the images or icons under our CC-BY 4.0 license. Alternatively, you may replace the images with open source alternatives. See these open source resources you may use to replace images / clip-art:

- https://commons.wikimedia.org

- https://openclipart.org/

If the reviewer comments include a recommendation to cite specific previously published works, please review and evaluate these publications to determine whether they are relevant and should be cited. There is no requirement to cite these works unless the editor has indicated otherwise.

Additional Editor Comments (if provided):

[Note: HTML markup is below. Please do not edit.]

Reviewers' comments:

Reviewer's Responses to Questions

**Comments to the Author**

1. Does this manuscript meet PLOS Global Public Health’s publication criteria? Is the manuscript technically sound, and do the data support the conclusions? The manuscript must describe methodologically and ethically rigorous research with conclusions that are appropriately drawn based on the data presented.

Reviewer #1: Partly

Reviewer #2: Partly

**********

2. Has the statistical analysis been performed appropriately and rigorously?

Reviewer #1: Yes

Reviewer #2: Yes

**********

3. Have the authors made all data underlying the findings in their manuscript fully available (please refer to the Data Availability Statement at the start of the manuscript PDF file)?

The PLOS Data policy requires authors to make all data underlying the findings described in their manuscript fully available without restriction, with rare exception. The data should be provided as part of the manuscript or its supporting information, or deposited to a public repository. For example, in addition to summary statistics, the data points behind means, medians and variance measures should be available. If there are restrictions on publicly sharing data—e.g. participant privacy or use of data from a third party—those must be specified.

Reviewer #1: Yes

Reviewer #2: Yes

**********

4. Is the manuscript presented in an intelligible fashion and written in standard English?

PLOS Global Public Health does not copyedit accepted manuscripts, so the language in submitted articles must be clear, correct, and unambiguous. Any typographical or grammatical errors should be corrected at revision, so please note any specific errors here.

Reviewer #1: Yes

Reviewer #2: Yes

**********

5. Review Comments to the Author

Please use the space provided to explain your answers to the questions above. You may also include additional comments for the author, including concerns about dual publication, research ethics, or publication ethics. (Please upload your review as an attachment if it exceeds 20,000 characters)

Reviewer #1: This manuscript addresses an important public health issue: the association between ambient temperature and ischemic heart disease mortality over a 22-year period in the United States. The large temporal scope and use of nationally representative datasets are strengths. The modeling approach (negative binomial regression with DLNM) is appropriate for aggregated mortality counts. However, substantial revisions are necessary.

First, the manuscript overstates attribution. No attributable fraction or excess mortality calculation is performed, yet the title and conclusions frame the findings as “population burden attributable to temperature.” This language must either be supported by formal burden estimation or removed.

Second, the claim that the model explains 93.6% of mortality variability is misleading. The deviance reduction reflects inclusion of strong seasonal and state fixed effects and should not be interpreted as temperature explaining nearly all variability.

Third, the ecological design limits causal inference. Strong preventive or causal language should be tempered, and ecological fallacy risk more thoroughly discussed.

Fourth, potential confounding by influenza, air pollution, and socioeconomic factors is not addressed beyond a brief limitation statement. Winter increases in cardiovascular mortality may partly reflect seasonal infections and pollution patterns.

Fifth, reliance on monthly mean temperature likely attenuates extreme heat effects. The absence of heat-associated mortality should be interpreted cautiously. Additionally, subgroup instability, particularly in small racial groups, also warrants careful qualification.

Therefore, to make it more didactic, I separate them into major and minor comments.

Major Comments:

1. The most striking finding is the "protective" effect of warmer monthly temperatures (RR 0.90). In environmental health, heat is typically associated with acute cardiovascular stress. The authors must explicitly state that monthly averages smooth out daily spikes. A month with a high average temperature might actually have fewer cold days (reducing IHD risk) but several dangerous heatwave days that are "lost" in the average. This distinction is vital for global public health policy to ensure that heat-action plans are not undermined by this data.

2. The use of state-level data is a significant limitation. States like California or New York contain multiple climate zones. Assigning a single average temperature to an entire state likely introduces measurement error. While the authors acknowledge this as a limitation, the Discussion should explore how this might bias the Relative Risk (RR) toward the null or create artificial associations.

3. The authors centered their model at 52.8°F (the mean). While statistically sound, usually temperature-mortality studies center at the Minimum Mortality Temperature (MMT). Since the MMT in this study was at the very high end of the distribution, the authors should explain the biological or public health implications of this—does it suggest that the U.S. population is better adapted to heat than cold, or is it a result of widespread air conditioning?

Minor Comments:

1. The lack of significance in the "Under 45" and "American Indian/Alaska Native" groups is attributed to low power/small counts. Please provide the raw number of deaths for these groups in the supplement to help the reader assess the degree of under-powering.

2. While year and state fixed effects are used, factors like PM2.5 (air pollution) often fluctuate with temperature and are independent risk factors for IHD. A brief discussion on how air quality might interact with these findings would strengthen the manuscript.

3. In Figure 1, the 3D surface plot is useful, but the contour lines in Figure 2 are more informative for policy. Ensure the resolution of Figure 1 is high enough to distinguish the "lag" axis clearly.

4. It is imperative that authors employ units of measurement that are consistent with the international system of units (SI). Consequently, the measurement of temperature could be expressed in degrees Celsius (°C).

In summary, the study has merit and potential impact, but major revision is required to align conclusions with the analytical scope, improve statistical interpretation, and strengthen methodological transparency.

Reviewer #2: 1. The introduction is comprehensive and well-written. However, the research gap is not well defined: monthly vs daily exposure is mentioned but not justified enough. The rationale for focusing on monthly mean temperature needs stronger methdological grounding.

2. While the data sources are robust and whidely used, the ecological nature of the analysis introduces important limitations, particularly the risk of ecological fallacy and potential misclassification of cause of death, which are currently under-discussed and may impact the validity and interpretation of the findings.

3. The statistical model adjusts only for state, year, and month, but does not account for important potential confounders such as air pollution, seasonal infections (e.g. influenza), socioeconomic status, and access to healthcare, which represents a major limitation and may significantly bias the obsesrved association between ambient temperature and mortality.

4. Although the results are clearly presented, the interpretation of the inverse temperature-mortality relationship as a ”protective effect of heat” is not justified, as the ecological study design does not support causal interference and the observed associationi may be influenced by residual confounding or methodological factors rather than a true protective effect.

5. The results are presented with excessive detail and repetition; for example, Table 1 and 3 repoert multiple similar relative risks across temperature percentiles and subgroups with minimal added insight, while Table 4 includes extensive model fit statistics that could be moved to the supplementary material to improve clarity.

6. The lag analysis is limited to a 3-month period without a clear methodological or biological justification, and the rationale for this lag structure should be better explained.

7. The interpratation of heat as ”protective” and cold as a causal factor is overstated, as the ecological design only supports associations and does not justify causal inference.

8. The comparison with existing literature is adequate, but the discussion remains superficial, particularly regarding the differences between studies using daily versus monthly temperature data, and would benefit from a more explicit explanation of why the present findings diverge from the commonly reported U of J-shaped relationships.

9. The interpretation of subgroup findings is occasionally too strong given the wide confidence intervals and instability of some estimates, and should be presented more cautiously with greater emphasis on uncertainty.

10. The figures are visually appropriate but insufficiently explaind. I recommend you to improve figure legends.

11. The reference list is generally appropriate, however, it includes several older studies, and would benefit from the inclusion of more recent research (post 2020?) to better reflect current evidence.

**********

6. PLOS authors have the option to publish the peer review history of their article (what does this mean?). If published, this will include your full peer review and any attached files.

**Do you want your identity to be public for this peer review?**  If you choose “no”, your identity will remain anonymous but your review may still be made public.

For information about this choice, including consent withdrawal, please see our Privacy Policy.

Reviewer #1: No

Reviewer #2: No

**********

[NOTE: If reviewer comments were submitted as an attachment file, they will be attached to this email and accessible via the submission site. Please log into your account, locate the manuscript record, and check for the action link "View Attachments". If this link does not appear, there are no attachment files.]

-->Figure Resubmissions:

While revising your submission, we strongly recommend that you use PLOS’s NAAS tool (https://ngplosjournals.pagemajik.ai/artanalysis) to test your figure files. NAAS can convert your figure files to the TIFF file type and meet basic requirements (such as print size, resolution), or provide you with a report on issues that do not meet our requirements and that NAAS cannot fix.

After uploading your figures to PLOS’s NAAS tool - https://ngplosjournals.pagemajik.ai/artanalysis, NAAS will process the files provided and display the results in the "Uploaded Files" section of the page as the processing is complete. If the uploaded figures meet our requirements (or NAAS is able to fix the files to meet our requirements), the figure will be marked as "fixed" above. If NAAS is unable to fix the files, a red "failed" label will appear above. When NAAS has confirmed that the figure files meet our requirements, please download the file via the download option, and include these NAAS processed figure files when submitting your revised manuscript.

Attachment Submitted filename: Reviewers Report PONE-D-25-63906.pdf

# Author comment: Author response to Decision Letter 1

**DOI:** 10.1371/journal.pgph.0006641.r002

8 Apr 2026

Attachment Submitted filename: Response to Reviewers.pdf

# Peer review: Decision Letter 1

**DOI:** 10.1371/journal.pgph.0006641.r003

**Authors:** Katrien Janin, Julia Robinson

27 May 2026

"The Association Between Monthly Ambient Temperatures and Ischemic Heart Disease Mortality in the United States: A Nationwide Ecological Analysis, 1999–2020"

PGPH-D-26-00370R1

Dear Dr Ahmed,

We are pleased to inform you that your manuscript '"The Association Between Monthly Ambient Temperatures and Ischemic Heart Disease Mortality in the United States: A Nationwide Ecological Analysis, 1999–2020" ' has been provisionally accepted for publication in PLOS Global Public Health.

Before your manuscript can be formally accepted you will need to complete some formatting changes, which you will receive in a follow up email. A member of our team will be in touch with a set of requests.

Please note that your manuscript will not be scheduled for publication until you have made the required changes, so a swift response is appreciated.

IMPORTANT: The editorial review process is now complete. PLOS will only permit corrections to spelling, formatting or significant scientific errors from this point onwards. Requests for major changes, or any which affect the scientific understanding of your work, will cause delays to the publication date of your manuscript.

If your institution or institutions have a press office, please notify them about your upcoming paper to help maximize its impact. If they'll be preparing press materials, please inform our press team as soon as possible -- no later than 48 hours after receiving the formal acceptance. Your manuscript will remain under strict press embargo until 2 pm Eastern Time on the date of publication. For more information, please contact globalpubhealth@plos.org.

Thank you again for supporting Open Access publishing; we are looking forward to publishing your work in PLOS Global Public Health.

Best regards,

Julia Robinson

Executive Editor

PLOS Global Public Health

***********************************************************

Reviewer Comments (if any, and for reference):

Reviewer's Responses to Questions

**Comments to the Author**

1. If the authors have adequately addressed your comments raised in a previous round of review and you feel that this manuscript is now acceptable for publication, you may indicate that here to bypass the “Comments to the Author” section, enter your conflict of interest statement in the “Confidential to Editor” section, and submit your "Accept" recommendation.

Reviewer #3: All comments have been addressed

**********

2. Does this manuscript meet PLOS Global Public Health’s publication criteria? Is the manuscript technically sound, and do the data support the conclusions? The manuscript must describe methodologically and ethically rigorous research with conclusions that are appropriately drawn based on the data presented.

Reviewer #3: Yes

**********

3. Has the statistical analysis been performed appropriately and rigorously?

Reviewer #3: Yes

**********

4. Have the authors made all data underlying the findings in their manuscript fully available (please refer to the Data Availability Statement at the start of the manuscript PDF file)?

The PLOS Data policy requires authors to make all data underlying the findings described in their manuscript fully available without restriction, with rare exception. The data should be provided as part of the manuscript or its supporting information, or deposited to a public repository. For example, in addition to summary statistics, the data points behind means, medians and variance measures should be available. If there are restrictions on publicly sharing data—e.g. participant privacy or use of data from a third party—those must be specified.

Reviewer #3: Yes

**********

5. Is the manuscript presented in an intelligible fashion and written in standard English?

PLOS Global Public Health does not copyedit accepted manuscripts, so the language in submitted articles must be clear, correct, and unambiguous. Any typographical or grammatical errors should be corrected at revision, so please note any specific errors here.

Reviewer #3: Yes

**********

6. Review Comments to the Author

Please use the space provided to explain your answers to the questions above. You may also include additional comments for the author, including concerns about dual publication, research ethics, or publication ethics. (Please upload your review as an attachment if it exceeds 20,000 characters)

Reviewer #3: It appears that the previous review elicited appropriate responses and revisions from the authors in that overstatement lead to “ burden- or attribution related terminology” which was removed. As is often the case with statistical presentations, it was imperative to accentuate associative findings rather than causal impact of some statements and one must Include residual confounding on the findings.

The modeling was not an issue. Appropriate inputs and interpretation had to be adjusted in several cases. Interpretation of null effects in this subgroup should be made cautiously as well as caution of over interpreting null effects in small samples.

Upon a read of the revision the modeling stood firm in that a statistical conclusion is that the use of negative binomial regression within a distributed lag non-linear model (DLNM) captured both non-linear and delayed effects of ambient temperature while minimizing overdispersion.

**********

7. PLOS authors have the option to publish the peer review history of their article (what does this mean?). If published, this will include your full peer review and any attached files.

**Do you want your identity to be public for this peer review?**  If you choose “no”, your identity will remain anonymous but your review may still be made public.

For information about this choice, including consent withdrawal, please see our Privacy Policy.

Reviewer #3: No

**********
